# Supplementary material for: Organic matter mineralization in modern and ancient ferruginous sediments
Source: Nat Commun. 2021 Apr 13;12:2216. doi: 10.1038/s41467-021-22453-0 (PMC8044167; doi:10.1038/s41467-021-22453-0)
Supplement: Supplementary file 1 — Supplementary Information [file 41467_2021_22453_MOESM1_ESM.pdf]

Supplementary Information

Organic matter mineralization in modern and ancient ferruginous sediments

André Friese, Kohen Bauer, Clemens Glombitza, Luis Ordoñez, Daniel Ariztegui, Verena B. Heuer, Aurèle Vuillemin, Cynthia Henny, Sulung Nomosatryo, Rachel Simister, Dirk Wagner, Satria Bijaksana, Hendrik Vogel, Martin Melles, James M. Russell, Sean A. Crowe*, Jens Kallmeyer*, and the Towuti Drilling Project Science Team

*Correspondence: jens.kallmeyer@gfz-potsdam.de, sean.crowe@ubc.ca

Materials and Methods

Pore water sampling and analysis

Whole round cores (WRC, 100 mm long x 66 mm diameter) were cut from the recovered sediment drill core, immediately capped and transferred into a N_2-filled anaerobic chamber that was set up on site. Sediment was transferred under N_2 to an IODP-Style PTFE-titanium pore water extractor [1] and squeezed using a 22-ton hydraulic press (Carver Inc., Wabash, USA). Pore water samples were filtered through a sterile 0.2 µm syringe filter and collected in a glass syringe that was pre-flushed with nitrogen. Dissolved Fe concentrations were analyzed on site whereas the remaining pore water samples were preserved for later analysis of dissolved cat- and anions as well as volatile fatty acids (VFAs).

Concentrations of major anions (i.e. Cl^-, SO_4^{2-}, NO_3^-, NO_2^-) in the pore water were measured by suppressed ion chromatography using a SeQuant SAMS anion IC suppressor (EMD Millipore, Billerica, Massachusetts), a S5200 sample injector, a 3.0 × 250 mm LCA 14 column and a S3115 conductivity detector (all Sykam, Fürstenfeldbruck, Germany). The eluent was 5 mM Na_2CO_3 with 20 mg L^{-1} 4–hydroxybenzonitrile and 0.2% methanol. Flow rate was set to 1 mL min^{-1} and column oven temperature to 50°C. For cations (i.e. Na^+, NH_4^+, K^+, Mg^{2+}, Ca^{2+}),

the IC system consisted of a S5300 sample injector (Sykam), a 4.6 × 200 mm ReprosilCAT column (Dr. Maisch HPLC, Ammerbuch-Entringen, Germany) and a S3115 conductivity detector (Sykam). The eluent was 175 mg L^{-1} 18-Crown-6 and 120 µL methanesulfonic acid. Flow rate was set to 1.2 mL min^{-1} and column oven temperature to 30°C. Detection and quantification limits were calculated based on signal-to-noise (S/N) ratios of 3 and 10, respectively. All samples were measured in triplicates and every ten injections a standard was measured to check for drift. Reproducibility was always better than 5% for each ion. Detection limits for the major an- and cations ranged between 1 – 4 µM and 6 – 11 µM, respectively.

The pH was measured with a portable pH meter (Thermo Scientific Orion, Star A321) calibrated at pH 4, 7 and 10, respectively. We homogenized 2 mL of sediment in 2 mL of deionized water and measured the supernatant after 2 min, according to EPA method 9045D [2]. Alkalinity was measured via colorimetric titration on a sample of hydraulically squeezed pore water. No replicates could be performed due to lack of available sediment and pore water but reproducibility was usually better than 10%. Dissolved inorganic carbon (DIC) concentrations were calculated by solving the carbonate system using the pH and alkalinity profiles and borehole temperatures.

Concentrations of volatile fatty acids (VFAs) in the pore water were measured by 2-dimensional ion chromatography mass spectrometry (2D IC-MS) [3]. This technique allows analysis of the following VFAs: lactate, acetate, propionate, formate, butyrate, pyruvate, valerate [3]. As the method was originally developed for marine pore water samples, some modifications were made, as described below, to account for the low salinity of the pore water of Lake Towuti. The instrument used for 2D IC-MS analysis was a Dionex ICS3000 coupled to a Surveyor MSQ Plus mass spectrometer (both Thermo Scientific). Briefly, in this method the first IC dimension is used to separate the VFAs from other inorganic ions. The VFAs are trapped on a concentrator column and subsequently separated in the second IC dimension. To account for the effect of low salinity, the retention time window of the eluent flow from the first column that is directed to the concentrator column was shifted by one minute to 3.5 - 8.5 min as compared to the marine pore water analysis protocol [3]. Prior to analysis, the samples were filtered through disposable Acrodisc® 13 mm IC syringe filters (pore size 0.2 µm) that were rinsed with 10 mL Milli-Q® water (Ultrapure Type 1) directly before use. The first 0.5 mL of pore water after filtration was discarded while the second 0.5 mL was used for analysis. Quantification was achieved by a 3-

point calibration with external standards containing a mixture of the analyzed VFAs at different concentrations (i.e. 200, 500 and 800 μg L^{-1}) prepared in Milli-Q® water. Blank runs of pure Milli-Q® water were used to correct the peak areas for the small background peaks deriving from accumulation of VFAs from the eluent in the trap column. Detection limits for the individual VFAs were all between 0.1 and 0.4 μM (i.e. formate: 0.37 μM, acetate: 0.19 μM, propionate 0.13 μM, butyrate: 0.09 μM).

TOC analysis

The total organic carbon (TOC) was analyzed by Rock-Eval 6 pyrolysis (Vinci Technologies). In the pyrolysis step ~ 60 mg sediment were heated to 650°C in an inert atmosphere. This released free hydrocarbons that were measured by a flame ionization detector (FID). Thermal cracking of long chain carbon compounds and carbonates produced CO and CO_2 that were measured simultaneously by an infrared-cell. The carbonate related peak could be accurately identified allowing the differentiation between mineral and organic carbon. In a second step the material was reheated to 850 °C to quantify the remaining refractory organic matter. TOC (%) was calculated according to [4].

Methane concentrations and isotopic analysis

For methane analysis, 2 cm^3 of sediment was retrieved with a cutoff syringe immediately after core retrieval and transferred to a 20 mL crimp vial filled with saturated NaCl solution and stored at 4°C without any headspace. Before analysis, 3 mL Helium was introduced as a headspace to all samples followed by equilibration for at least 24 hours. Methane concentrations were determined by injecting 200 μL of the He headspace into a Thermo Finnigan Trace gas chromatograph equipped with a flame ionization detector (Thermo Fisher Scientific). Helium was used as a carrier gas with a constant flow rate of 2 mL min^{-1} and the split ratio was set to 5.

In the 12 m core we analyzed the isotopic composition of the pore water methane and CO_2. $\delta^{13}C(CH_4)$, $\delta^{13}C(CO_2)$ and $\delta D(CH_4)$ were determined by injecting He headspace to a continuous-flow isotope ratio mass spectrometer (Delta V Plus, Thermo Fisher Scientific) equipped with a Trace GC Ultra (Thermo Fisher Scientific). For $\delta^{13}C(CH_{4,})$ and $\delta^{13}C(CO_2)$ injection volume was 200 μL. Flow rate of the carrier gas was 3 mL min^{-1} and the split ratio was set to 3. For $\delta D(CH_4)$ 400 μL of He headspace was injected. Flow rate was 2 mL min^{-1} and the split ratio was 5. Results are in δ notation: $\delta^{13}C = ([^{13}C/^{12}C]_{sample}/[^{13}C/^{12}C]_{standard} - 1)*1000$ ‰

referenced against Vienna Pee Dee Belemnite (VPDB, $^{13/12}C$ ratio 0.0112372 ±0.0000090) and $\delta D = ([^2H/^1H_{sample}]/[^2H/^1H_{standard}] - 1)*1000$ ‰ referenced against Vienna Standard Mean Ocean water (VSMOW, $^{2/1}H$ ratio 0.00015576 ± 0.00000010). Due to limited sample volume, duplicates could only be measured on select samples. Reproducibility was always better than 5%.

Potential sulfate reduction rates

All incubations were done in triplicate. The microbially produced TRIS (total reduced inorganic sulfur) species were separated from the remaining sample and the unreacted sulfate tracer using the cold chromium distillation of Kallmeyer et al. (2004) [5]. We added 8 mL 6N HCl, 16 mL of 1 M $CrCl_2$ and 20 mL of dimethylformamide to the distillation flask. Prior to addition of the reagents a small amount of non-radioactive zinc sulfide suspension was added as a sulfide carrier to enhance recovery. The released H_2S was collected in a trap filled with 5 % zinc acetate solution where H_2S was precipitated as zinc sulfide. Fresh traps were used for each fraction. The zinc acetate solution was transferred to a scintillation vial, then 8 mL Ultima Gold Scintillation Cocktail (Perkin Elmer) was added and the mixture homogenized. The radioactivity of recovered sulfide was quantified using a Tri Carb 2500 TR liquid scintillation counter (Packard Instruments, Meriden, CT, USA). Potential sulfate reduction rates were quantified according to [6] with the following equation:

$$\mathrm{SRR} = [\mathrm{SO_4^{2-}}] \times P_{\mathrm{SED}} \times \frac{a_{\mathrm{TRIS}}}{a_{\mathrm{TOT}}} \times \frac{1}{t} \times 1.06 \times 10^6 \qquad (1)$$

where SRR is the sulfate reduction rate (pmol $cm^{-3}d^{-1}$); $[SO_4^{2-}]$ the sulfate concentration in the pore water (mmol L^{-1}) plus 0.01 mmol L^{-1} non-radioactive sulfate that was added to the radiotracer; P_{SED} is the sediment porosity (mL pore water cm^{-3} sediment); a_{TRIS} is the radioactivity of TRIS (counts per minute); a_{TOT} is the total radioactivity used (counts per minute); 1.06 is the correlation factor for the expected isotopic fractionation [7] and 10^6 is the factor for the change of units from mmol cm^{-3} d^{-1} to pmol cm^{-3} d^{-1}. During incubation, turnover of the injected radiotracer was always below 1 % in all experiments. The depth-integrated potential sulfate reduction rate (mmol m^{-2} yr^{-1}) was calculated as the sum of all measured mean potential sulfate

reduction rates from 0 to 12 m. The uncertainty of that rate is the sum of the respective standard deviations.

Geochemical modeling

Net reaction rates of dissolved chemical species linked to select microbial metabolisms (i.e. Fe^{2+}, CH_4, NH_4^+,VFAs) were calculated using the MATLAB script of Wang et al. (2008) [8], assuming that the pore water concentration profiles represent steady-state conditions. We used a measured porosity profile (Table S1), which the model requires to calculate the formation factor based on the empirical relationship $f = 10.0196\ \phi^{-1.8812}$. The model applied a 5-point Gaussian filter to the respective pore water concentration profile, formation factor and porosity. Diffusion coefficients of the respective compounds were obtained from the compilation of [9] and were corrected for in-situ temperature using a temperature profile that was obtained by downhole logging (Table S1). We used a constant sedimentation rate of $1.9\ 10^{-4}$ m yr^{-1} [10] and a constant external flow advection velocity near the SWI. A minimum of three measured concentration data points was used to determine each reaction zone. The model quantifies uncertainties in the rate estimates by using a Monte Carlo technique [8].

Results of modeled turnover rates in the upper 0.5 m should usually be treated with caution because processes other than molecular diffusion can affect the pore water concentration gradients near the SWI. For example, bioturbation and/or advective transport can enhance the exchange of solutes between sediment and the overlying water [11,12], although we can preclude bioturbation in this particular case because of the anoxic bottom water.

Moreover, disturbances can be caused by the impact of the coring device, especially larger corers like we used for the 12 m core completely destroy the uppermost sediment layer. We thus retrieved a short core (< 0.4 m) with a small gravity corer that recovers an undisturbed SWI. This way we could increase the sampling resolution in the uppermost sediment layers, especially for methane. Due to the very steep gradient and strong curvature we combined the concentration profiles of both cores and used them as a single input for the model. Additionally, we ran a separate model for the short gravity core (Fig. S4). Both models are in good agreement to each other.

Summing up all modeled mean reaction rates within the upper 12 m yielded the depth-integrated reaction for the studied depth interval (mmol m^{-2} yr^{-1}). The sum of the respective

modeled standard deviations yielded the uncertainty of that rate. Using the data from our sequential Fe-speciation extraction we calculated depth-integrated rates of microbial Fe reduction by combining sedimentation rates with changes in Fe speciation between depth intervals, assuming steady-state deposition. We focused on sediment intervals in which a decrease in the total Fe(III) pool could be matched to a corresponding increase in the total Fe(II) pool, recognizing that some Fe(II) can be lost to the overlying water column via diffusive transport.

Potential methane production

We performed incubation experiments with sediment samples from three different depths (0.36 m, 1.95 m and 7.4 m) to investigate the potential for methane production in Lake Towuti sediment. Using a sterile cutoff syringe, we retrieved sediment samples of 0.5 cm^3 each from WRCs that were stored in nitrogen-filled aluminum foil bags at room temperature. The respective sediment sample was transferred into an autoclaved 5 mL glass crimp vial together with 1 mL of sulfate-depleted freshwater medium mimicking the pore water concentrations of Lake Towuti sediment (Table S2). Due to the low pore water sulfate concentrations, we substituted the regular sulfur-containing reducing agent (sodium sulfide) with titanium citrate with a final concentration of 1.6 mM in the medium. To check for the potential of hydrogenotrophic methanogenesis, the butyl stoppered glass crimp vial was flushed with a mixture of H_2/CO_2 (80/20 %). A second experiment was set up, in which acetate was added with a final concentration of 100 µM in the medium and N_2/CO_2 (80/20 %) in the headspace. For controls the respective sediment samples were mixed with medium (Table S2) and the headspace flushed with N_2/CO_2 (80/20 %). Killed controls were not run. All sample handling was done in a nitrogen-filled anaerobic chamber. The vials were constantly shaken in the dark at 100 rpm at a constant temperature of 28°C. Methane production in the experiments was measured by gas chromatography (Agilent Technologies, CA, USA).

After 35 days of incubation, headspace methane concentrations in the hydrogenotrophic incubations reached 17, 5.9 and 2.8 vol. % in sediment from 0.36, 2 and 7.4 m, respectively (Fig. S2a). Acetoclastic methane production showed the same depth trend, albeit at a lower rate, never exceeding methane concentrations of 3 vol. % (Fig. S2b). This is in line with the distribution of methane production indicated by modeling pore water profiles and methane isotopic

compositions (Fig. 3 & 4). In unamended control experiments methane production could only be observed in the uppermost sample and did not reach more than 1 vol. %, implying that rates of methane production are limited by H_2 and acetate substrate supply rates.

Methane production and accumulation in the oceans and atmosphere

Solutions to photochemical models [13] run across a range of biospheric CH_4 fluxes can be approximated by the following equation [14]:

$$f(CH_4) = a \times flux_{CH4}^{b} \qquad [1]$$

Where $f(CH_4)$ is the atmospheric CH_4 mixing ratio, a (=1.474×10^{-26}) and b (=2.0291) are tunable constants, and $flux_{CH4}$ is the biospheric CH_4 flux (molecules cm^{-2} s^{-1}).

Methane fluxes were estimated by considering global organic matter mineralization of 36-396 Tmol yr^{-1} and a 2:1 stoichiometry for the production of methane by organic carbon degradation according to the reaction:

$$2CH_2O \rightarrow CH_4 + CO_2 \qquad [2]$$

In reality, this reaction proceeds through a number of fermentation steps, but the overall stoichiometry remains the same. We then considered that in the low Fe(III) reactivity end member 8-15% of this 36-396 Tmol yr^{-1} CH_2O was first degraded by Fe(III) reduction, which left 30-364 Tmol yr^{-1} CH_2O to fuel methanogenesis. Based on 2:1 stoichiometry, this translates to global methane production fluxes of 15-182 Tmol yr^{-1}. Likewise, with 80% CH_2O degradation channeled through Fe(III) reduction in the high reactivity end member, 7.2-80 Tmol yr^{-1} CH_2O remains to fuel methanogenesis for 4-40 Tmol yr^{-1} global methane production, considering the stoichiometry mentioned above.

Supplementary Table 1: Input parameters for geochemical modeling. Input parameters for modeling of net turnover rates after the MATLAB script of Wang et al. (2008).

SEPARATE XLS FILE

Supplementary Table 2: Medium composition for potential methane production experiments. Medium composition for incubation of methanogens in Lake Towuti sediment.

**Compound**	**Concentration (mg L^{-1})**
$MgCl_2*6H_2O$	54
$CaCl_2*2H_2O$	16
NH_4Cl	5
K_2HPO_4	1.7
$NaHCO_3$	500
$FeCl_2 * 4H_2O$	7.5
$CoCl_2 * 6H_2O$	0.95
$MnCl_2*4H_2O$	0.5
$ZnCl_2$	0.35
$N_2MoO_4*2H_2O$	0.18
$NiCl_2*6H_2O$	0.12
H_3BO_3	0.03
$CuCl_2*2H_2O$	0.01
4-Aminobenzoate	0.1
Nicotinic acid	0.1
Ca-Pantothenate	0.1
Pyridoxine-Hydrochloride B6	0.1
Riboflavin	0.1
Thyamine-Hydrochloride	0.1
Biotin	0.05
Folic acid	0.05
Lipoic acid	0.05
Vitamine B12	0.05
Titanium citrate	237
Na-Resazurine	0.5

Supplementary Table 3: Modeled Saturation indices. Values were modeled with PHREEQC v.3 based on pH, alkalinity, pore water concentrations of major ions and borehole temperatures (modified after Vuillemin et al., 2019.

5 m: zone 1	Saturation	10 m: zone 2	Saturation	35 m: zone 4	Saturation
talc	1.43	siderite	1	siderite	1
siderite	1.29	quartz	0.71	quartz	0.71
quartz	0.71	chalcedony	0.29	chalcedony	0.29
chalcedony	0.29	vivianite	-0.04	vivianite	-0.04
vivianite	-0.45	talc	-0.31	talc	-0.31
α SiO_2	-0.54	α SiO_2	-0.54	α SiO_2	-0.54
calcite	-0.68	calcite	-0.83	calcite	-0.83
dolomite	-0.77	aragonite	-0.97	aragonite	-0.97
aragonite	-0.82	dolomite	-1.27	dolomite	-1.27

Supplementary Table 4: Compilation of turnover rates for reactants involved in organic matter mineralization. Rates were calculated for individual depth intervals, given as the mean rate as calculated by the model [52] and plus or minus one standard deviation.

SEPARATE XLS FILE

Supplementary Table 5: Concentration of the individual fractions of the sequential Fe extractions. Values are given as minimum/average/maximum estimates.

SEPARATE XLS FILE

Supplementary Table 6: Iron concentrations measured by quantitative XRD (qXRD) and dithionite extractions. The data show that within error of the qXRD and Fe-speciation measurements, dithionite quantitatively extracts goethite in the Lake Towuti sediments.

		Depth Interval (1-2 cm)			Depth Interval (35-40 cm)		
Mineral	Ideal Formula	Mass (wt%)	XRD Fe (wt%)	Fe-speciation (wt%)	Mass (wt%)	XRD Fe (wt%)	Fe-speciation (wt%)
Goethite	α-FeO(OH)	9.0	5.7	4.8	7.0	4.4	3.7

Supplementary Figure 1: Potential methane production experiments. Incubation experiments for potential hydrogenotrophic methanogenesis (A) and acetoclastic methanogenesis (B). Samples came from depths of 0.36 m, 1.95 m and 7.4 m, indicated in pink, green and yellow, respectively. Circles are experiments with added substrate H_2/CO_2 (80/20%) in the headspace (A) or 100 µM acetate in the medium (B). Triangles are negative controls with N_2/CO_2 (80/20%) headspace. The negative controls were not amended with substrate, but not killed.

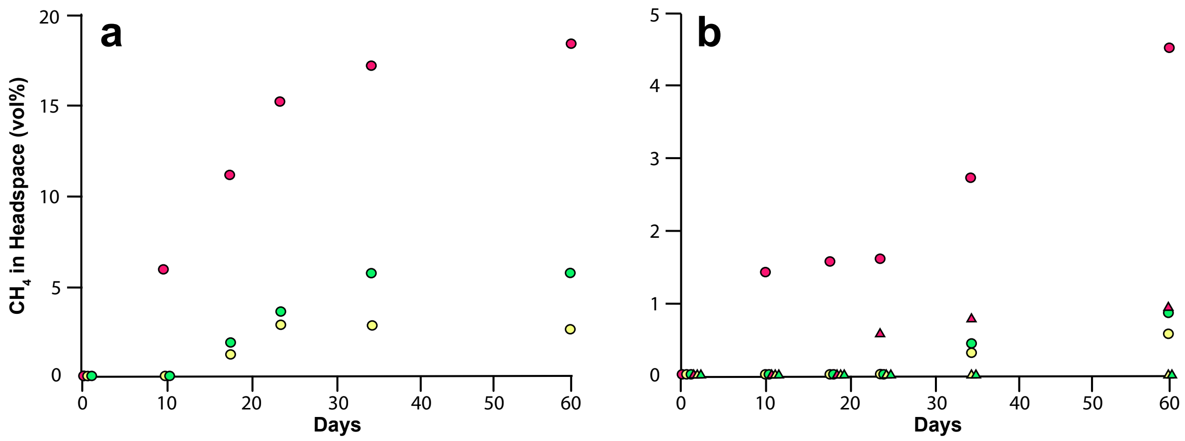

Supplementary Figure 2: Geochemical modeling of pore water sulfate. Geochemical modeling of net turnover rates of pore water sulfate for the 12 m drill core. The modeled turnover rates are 10 – 160 times lower than the measured potential sulfate reduction rates, which is consistent with previous observations on Lake Towuti sediments and similar environments [15-17]

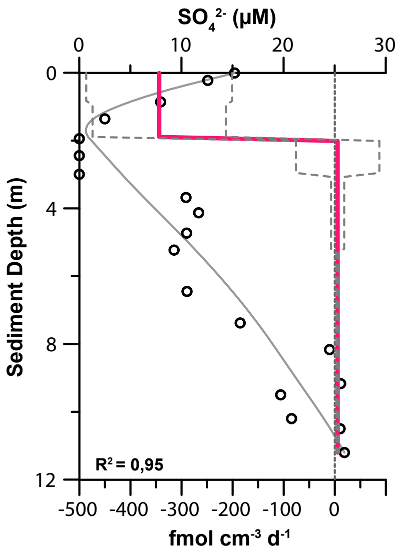

Supplementary Figure 3: Geochemical modeling of pore water methane. Geochemical modeling of net turnover rates of pore water methane for the short core (<0.4 m) that was retrieved with a gravity coring device to better preserve the sediment water interface and thus to increase the accuracy of the modeling results. The net turnover rates show a good fit with those of our model in Figure 2 and even predict methane production rates up to 9 µmol cm^{-3} d^{-1} in the upper 3 cm of the sediment core yielding a depth integrated methane production rate of 162 ± 19 mmol m^{-2} yr^{-1} for the upper 0.4 m. Please note that the methane concentration in the uppermost sample (0-0.5 cm) is not zero but around 10 µM, indicating a net flux of methane out of the sediment.

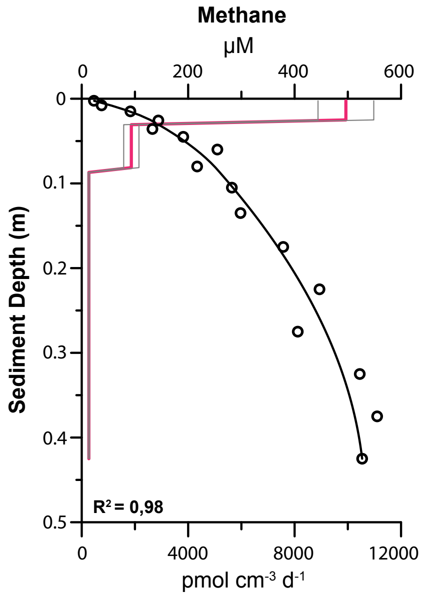

Supplementary Figure 4: XRD spectra for the upper 12 m of Lake Towuti sediment. The main reflectance peaks for goethite are identified (labeled vertical grey lines "G"), which reveal goethite in every sample.

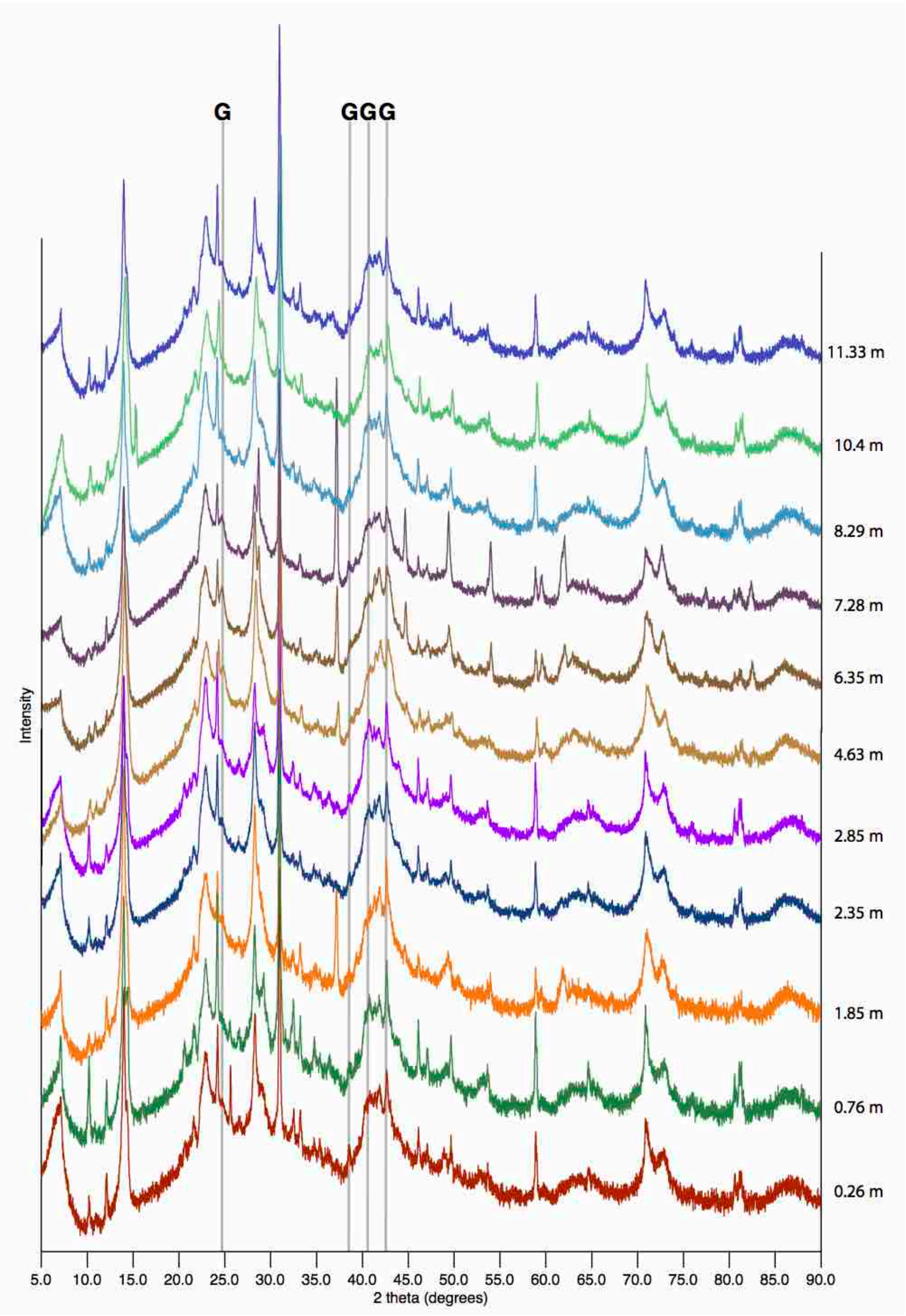

Supplementary References

1 Manheim, F. T. A hydraulic squeezer for obtaining interstitial water from consolidated and unconsolidated sediments. USGS Reference Paper 550, 171–174 (1966).
2 EPA. (EPA, 2004).
3 Glombitza, C., Pedersen, J., Røy, H. & Jørgensen, B. B. Direct analysis of volatile fatty acids in marine sediment porewater by two-dimensional ion chromatography-mass spectrometry. Limnology and Oceanography: Methods 12, 455-468 (2014).
4 Lafargue, E., Marquis, F. & Pillot, D. Rock-Eval 6 applications in hydrocarbon exploration, production, and soil contamination studies. Revue de l'institut français du pétrole **53** (1998).
5 Kallmeyer, J., Ferdelman, T. G., Weber, A., Fossing, H. & Jørgensen, B. B. A cold chromium distillation procedure for radiolabeled sulfide applied to sulfate reduction measurements. Limnology and Oceanography: Methods **2**, 171-180 (2004).
6 Jørgensen, B. B. A Comparison of Methods for the Quantification of Bacterial Sulfate Reduction in Coastal Marine Sediments 1. Measurement with radiotracer techniques. Geomicrobiol. J. **1**, 11-27 (1978).
7 Jørgensen, B. B. & Fenchel, T. The Sulfur Cycle of a Marine Sediment Model System. Marine Biology **24**, 189-201 (1974).
8 Wang, G., Spivack, A. J., Rutherford, S., Manor, U. & D'Hondt, S. Quantification of co-occurring reaction rates in deep subseafloor sediments. Geochimica et Cosmochimica Acta **72**, 3479-3488 (2008).
9 Schulz, H. D. in Marine Geochemistry (eds H. D. Schulz & M. Zabel) (Springer, 2000).
10 Russell, J. M. et al. Glacial forcing of central Indonesian hydroclimate since 60,000 y BP. Proceedings of the National Academy of Sciences of the United States of America **111**, 5100-5105, doi:10.1073/pnas.1402373111 (2014).
11 Ziebis, W., Forster, S., Huettel, M. & Jorgensen, B. B. Complex burrows of the mud shrimp Callianassa truncata and their geochemical impact in the sea bed. Nature **382**, 619-622, doi:10.1038/382619a0 (1996).
12 Precht, E. & Huettel, M. Rapid wave-driven advective pore water exchange in a permeable coastal sediment. Journal of Sea Research **51**, 93-107 (2004).
13 Pavlov, A. A., Brown, L. L. & Kasting, J. F. UV shielding of NH3 and O2 by organic hazes in the Archean atmosphere. Journal of Geophysical Research: Planets **106(E10)**, 23267-23287 (2001).
14 Ozaki, K., Tajika, E., Hong, P. K., Nakagawa, Y. & Reinhard, C. T. Effects of primitive photosynthesis on Earth's early climate system. Nature Geoscience **11**, 55 (2018).
15 Norði, K. A., Thamdrup, B. & Schubert, C. J. Anaerobic oxidation of methane in an iron-rich Danish freshwater lake sediment. Limnology and Oceanography **58**, 546-554 (2013).
16 Vuillemin, A. et al. Geomicrobiological features of ferruginous sediments from Lake Towuti, Indonesia. Frontiers in Microbiology **7**, doi:10.3389/fmicb.2016.01007 (2016).
17 Urban, N., Brezonik, P., Baker, L. & Sherman, L. Sulfate reduction and diffusion in sediments of Little Rock Lake, Wisconsi. Limnology and Oceanography **39**, 797-815.
